# Multimodal intrathecal analgesia (MITA) with morphine for reducing postoperative opioid use and acute pain following hepato-pancreato-biliary surgery: A multicenter retrospective study

**DOI:** 10.1371/journal.pone.0291108

**Published:** 2023-09-08

**Authors:** Vidhura Ratnasekara, Laurence Weinberg, Samuel Anthony Johnston, Luke Fletcher, Patrick Nugraha, Daniel Robert Anthony Cox, Raymond Hu, Ilonka Meyer, Osamu Yoshino, Marcos Vinius Perini, Vijayaragavan Muralidharan, Mehrdad Nikfarjam, Dong-Kyu Lee

**Affiliations:** 1 Department of Anaesthesia, Austin Health, Heidelberg, Australia; 2 Department of Critical Care, The University of Melbourne, Austin Health, Heidelberg, Australia; 3 Data Analytics Research and Evaluation (DARE) Centre, Austin Health, Heidelberg, Australia; 4 Department of Surgery, Austin Health, The University of Melbourne, Melbourne, Australia; 5 Department of Anesthesiology and Pain Medicine, Dongguk University Ilsan Hospital, Goyang, Republic of Korea; Asan Medical Center, University of Ulsan College of Medicine, REPUBLIC OF KOREA

## Abstract

**Introduction:**

The optimal analgesic modality for patients undergoing hepato-pancreato-biliary (HPB) surgery remains unknown. The analgesic effects of a multimodal intrathecal analgesia (MITA) technique of intrathecal morphine (ITM) in combination with clonidine and bupivacaine compared to ITM alone have not been investigated in these patients.

**Methods:**

We performed a multicenter retrospective study of patients undergoing complex HPB surgery who received ITM, bupivacaine, and clonidine (MITA group) or ITM-only (ITM group) as part of their perioperative analgesia strategy. The primary outcome was the unadjusted oral morphine equivalent daily dose (oMEDD) in milligrams on postoperative day 1. After adjusting for age, body mass index, hospital allocation, type of surgery, operation length, and intraoperative opioid use, postoperative oMEDD use was investigated using a bootstrapped quantile regression model. Other prespecified outcomes included postoperative pain scores, opioid-related adverse events, major complications, and length of hospital stay.

**Results:**

In total, 118 patients received MITA and 155 patients received ITM-only. The median (IQR) cumulative oMEDD use on postoperative day 1 was 20.5 mg (8.6:31.0) in the MITA group and 52.1 mg (18.0:107.0) in the ITM group (P < 0.001). There was a variation in the magnitude of the difference in oMEDD use between the groups for different quartiles. For the MITA group, on postoperative day 1, patients in the 25^th^ percentile required 14.0 mg less oMEDD (95% CI: -25.9 to -2.2; P = 0.025), patients in the 50^th^ percentile required 27.8 mg less oMEDD (95% CI: -49.7 to -6.0; P = 0.005), and patients in the 75^th^ percentile required 38.7 mg less oMEDD (95% CI: -72.2 to -5.1; P = 0.041) compared to patients in the same percentile of the ITM group. Patients in the MITA group had significantly lower pain scores in the postoperative recovery unit and on postoperative days 1 to 3. The incidence of postoperative respiratory depression was low (<1.5%) and similar between groups. Patients in the MITA group had a significantly higher incidence of postoperative hypotension requiring vasopressor support. However, no significant differences were observed in major postoperative complications, or the length of hospital stay.

**Conclusion:**

In patients undergoing complex HPB surgery, the use of MITA, consisting of ITM in combination with intrathecal clonidine and bupivacaine, was associated with reduced postoperative opioid use and resulted in superior postoperative analgesia without risk of respiratory depression when compared to patients who received ITM alone. A randomized prospective clinical trial investigating these two intrathecal analgesic techniques is justified.

## Introduction

Hepato-pancreato-biliary (HPB) surgery is commonly performed to resect benign and malignant lesions of the liver, pancreas, and gallbladder [1,2]. Such surgeries are often prolonged, with reported mortality rates of up to six percent in patients with multiple comorbidities [2]. Furthermore, laparotomy and extensive abdominal dissection can result in considerable postoperative pain [3]. Enhanced recovery after surgery (ERAS) programs have been used to reduce the onset of postoperative complications [4,5]. A major component of ERAS programs is optimal pain management in the perioperative period [6]. Acute pain is a major contributor to acute physiological stress following complex surgery and thus impacts the risks of complications in major organ systems [7]. Therefore, understanding how effective perioperative analgesia impacts complication rates and the length of hospital stay is of paramount importance [8].

Intrathecal morphine (ITM) has emerged as an effective analgesic technique for patients undergoing complex surgery and has been strongly advocated by the ERAS Society [9]. ITM use results in comparable postoperative pain control compared to epidural analgesia up to 48 hours after the completion of surgery, with no difference in complication rates [10,11]. However, the limitations of ITM use as monotherapy include delayed onset of analgesia and delayed respiratory depression [12]. The peak analgesic effect of ITM has been reported to occur approximately 6 hours after administration [12,13]. When compared to ITM alone, patients receiving ITM with bupivacaine required less morphine postoperatively, especially in the first 18 hours after surgery [12]. These improvements were associated with a small increase in postoperative hypotension, whereas other complications remained comparable [12,14].

Intrathecal analgesia using agents other than morphine has been well-described. Intrathecal clonidine, an alpha-2-adrenoceptor agonist, can be safely used without evidence of neurotoxicity and is frequently used together with local anesthetic agents to prolong their motor and sensory analgesic effects [15,16]. However, intrathecal clonidine has not been systematically investigated in patients undergoing complex HPB surgery or in combination with ITM in this setting. While the use of intrathecal clonidine in this context may have analgesic benefits, this combination of intrathecal agents may increase the incidence of postoperative hypotension and sedation [16,17].

To investigate the clinical benefits of this multimodal intrathecal technique, we conducted a retrospective analysis of patients who underwent complex HPB surgery and received multimodal intrathecal analgesia (MITA) - consisting of ITM in combination with intrathecal clonidine and bupivacaine, and compared it to ITM alone. Patients undergoing these surgeries across three teaching hospitals in metropolitan Melbourne were included to assess differences in postoperative opioid use, pain scores, and complications.

We hypothesized that MITA would be associated with reduced postoperative opioid requirements and pain scores without increasing respiratory depression, the development of major complications, or the length of hospital stay.

## Methods

### Study methodology

This retrospective multicenter observational study was conducted at three Australian hospitals. The Austin Hospital is a quaternary referral public hospital specializing in complex HPB surgeries, including liver transplantation. Warringal Private Hospital and Knox Private Hospital are private teaching hospitals that undertake complex HPB surgery. All three hospitals were served by hepatologists, perioperative physicians, HPB surgeons, anesthesiologists, and intensivists. All patients were managed using the same perioperative protocols and guidelines.

The key timelines for the study are as follows: i) 22/03/2022 –study protocol approved by the Austin Health Research Ethics Committee (approval number 2022/Austin/34); ii) 23/04/2022 –data collection following ethics approval; iii) 09/12/2022 –data collection completed; iv) 19/12/2022 –retrospectively registered with the Australian New Zealand Clinical Trials Registry (ACTRN12622001567718). Given that this was a retrospective observational study, registration of this study was undertaken after ethics approval and data collection. There were no changes to the original study protocol at any stage. Data analysis was only undertaken after trial registration. The authors confirm that all ongoing and related trials for this drug and intervention are registered. The study was conducted in accordance with the Strengthening the Reporting of Observational Studies in Epidemiology (STROBE) guidelines [18].

### Inclusion and exclusion criteria

Patients who underwent complex HPB surgery between January 2010 and June 2021 were identified using the hospital’s electronic health record. These patients were screened by two investigators between April 2022 and June 2022. Patients were included in the study if they were 18 years or older, underwent complex HPB surgery, and received either ITM alone or ITM in combination with intrathecal clonidine and bupivacaine.

Complex HPB surgery was defined as: liver surgery including excision of liver lesions, segmentectomy, or lobectomy; or pancreatic surgery including pancreaticoduodenectomy (Whipple procedure), total pancreatectomy, central pancreatectomy, and distal pancreatectomy with or without splenectomy. We excluded patients receiving epidural analgesia, those undergoing liver or pancreatic surgery secondary to other procedures, and those with a history of chronic pain syndromes or chronic opioid use (>60 mg oral morphine equivalent daily). The authors did not have access to identifying information during or after the data collection.

### Standardization of perioperative care

All patients across the three hospital sites underwent routine preoperative assessment, including cardiorespiratory status optimization. Hemoglobin optimization and transfusion practices were also undertaken according to the National Blood Authority of Australia’s patient blood management guidelines [19]. Postoperative management included mandatory ICU admission for at least one night, followed by discharge to a specialized HPB surgical ward that was led by a team consisting of a HPB surgeon, anesthesiologists, pain clinicians, and perioperative physicians.

After establishing intravenous (IV) access and standard hemodynamic monitoring, intrathecal analgesia was administered in the sitting position using a 25- or 27-gauge Quincke needle (Yale TM Spinal Becton Dickinson, Madrid, Spain) at the L3/4 or L4/5 interspace. All patients received intrathecal morphine sulfate (500 μg/1 mL solution) (Medicianz Healthcare Pty Ltd, Australia) alone, or in combination with 0.5% hyperbaric bupivacaine hydrochloride injection (Pfizer Pty Ltd, Perth, Australia) and clonidine hydrochloride injection (150 μg/1 mL solution) (Medicianz Healthcare Pty Ltd, Australia). The doses were determined at the discretion of the treating anesthesiologist.

Following intrathecal analgesia, patients who received MITA were placed in a 30-degree Trendelenburg position with flexion of their hips to increase the cephalad spread of the hyperbaric spinal anesthetic solution. Patients in the ITM group were placed supine, i.e., in a 0-degree or level position. Anesthesia was then induced using a standard balanced anesthesia induction technique. After an approximately 30-minute duration, patients in the MITA group were repositioned back to the level position.

All patients received an ERAS protocol for liver and pancreatic surgeries, as previously reported by Cosic et al. [20]. Arterial lines and central venous catheters were used for invasive monitoring in all patients. Blood products and fluids were administered according to hospital guidelines when clinically indicated. Vasoactive medications, including metaraminol and norepinephrine, were administered at the anesthesiologist’s discretion to maintain a mean arterial pressure greater than 65 mmHg.

### Predefined outcome variables

The primary outcome was the unadjusted postoperative analgesic use, defined as the cumulative oral morphine equivalent daily dose (oMEDD) in milligrams (mg) on postoperative day 1. The secondary outcomes included postoperative oMEDD adjusted for age, body mass index, hospital allocation, type of surgery, operation length, and intraoperative opioid use. Postoperative pain scores at rest and movement on postoperative days 0, 2, and 3, postoperative opioid-related adverse events, major postoperative complications, and length of hospital stay, were also evaluated.

Postoperative doses of opioids were converted to an equivalent dose of oral morphine using the Opioid Dose Equivalence Statement by the Faculty of Pain Medicine at the Australian and New Zealand College of Anaesthetists (ANZCA) [21]. Pain scores were measured using the Numerical Rating Scale (NRS), an 11-point numerical scale that defines a respondent’s severity of perceived pain between no pain designated a score of 0 and the worst possible pain designated a score of 10. Sedation scores were defined on a 4-point scale as follows: 0 = awake and alert, 1 = occasionally drowsy, easily roused, 2 = often drowsy, easily roused with touch, 3 = somnolent, and difficult to rouse. Complications were defined according to the European Perioperative Clinical Outcome definitions [22]. Complications were graded according to the Clavien-Dindo classification system [23]. Length of hospital stay was defined as the number of days between the completion of surgery and the patient’s discharge from the hospital.

### Data collection

Preoperative data were extracted from the electronic health records of each hospital by two investigators. The demographic data collected included age, sex, and body mass index (BMI). Preoperative data collected included principal diagnosis, operative procedure, major or minor surgery for liver resections, operative technique, and consultant surgeon.

Intraoperative data collected included the operation time and type of resection, including the number of segments resected for relevant liver surgeries. Anesthetic information included the dose of ITM, clonidine, and bupivacaine administered. Fluid observations, including urine output and blood loss, were collected. The amounts of intraoperative opioids, norepinephrine, and ephedrine were also recorded.

Postoperative data included maximum resting and movement pain scores and opioid consumption on postoperative days 0–3. Opioid-specific adverse events, including sedation, hypotension, and respiratory depression, were also collected on postoperative days 0–3. The lengths of ICU and hospital stays were recorded.

### Statistical analysis

Data analysis was performed using R v4.2.0 (R Core Team (2022). R: Language and environment for statistical computing. R Foundation for Statistical Computing, Vienna, Austria. URL https://www.R-project.org/) and the associated packages. The dataset was analyzed for missing data to assess whether this affected the significance of the associated statistical analysis. Appropriate statistical adjustments were applied according to the degree and type of missing data. Continuous variables were tested for normality using the graphical method of a quantile-quantile plot.

To investigate the association between patient characteristics and postoperative outcomes in the MITA and ITM groups, we used the Wilcoxon–Mann–Whitney test for continuous variables, and Fisher’s exact test or chi-squared test for categorical variables. All calculated P values were two-sided. We considered a two-tailed P value below 0.05 as indicative of statistical significance.

A boxplot was used to highlight the difference in oMEDD (mg) usage, pain scores at rest, and pain scores on movement in the postoperative period between the groups. This was calculated for days 0, 1, 2, and 3 in the postoperative period. The Wilcoxon–Mann–Whitney test was used to compare the statistical significance between the groups in their respective time periods.

To investigate oMEDD (mg) usage in the postoperative period, we used bootstrapped quantile regression to investigate the associated adjusted difference in oMEDD usage between the groups. oMEDD use was used as the dependent variable, and allocation to either the MITA or ITM group was used as the independent variable. Age (years), body mass index (BMI), hospital allocation, operation type (major liver, minor liver, major pancreatic, other pancreatic resection), operation length (min), and intraoperative IV morphine equivalent (mg) were a priori selected covariates. Quantile regression models the association between a set of input variables and specific percentiles (or quantiles) of the outcome variable and estimates differences in the quantiles of the outcome variable between MITA and ITM groups. We included three quantile regression models: 25th, 50th (median), and 75th percentiles. This was calculated on days 0–2 in the postoperative period. The full data sheet is available as a (S1 File).

## Results

### Patient cohort

**Fig 1:**
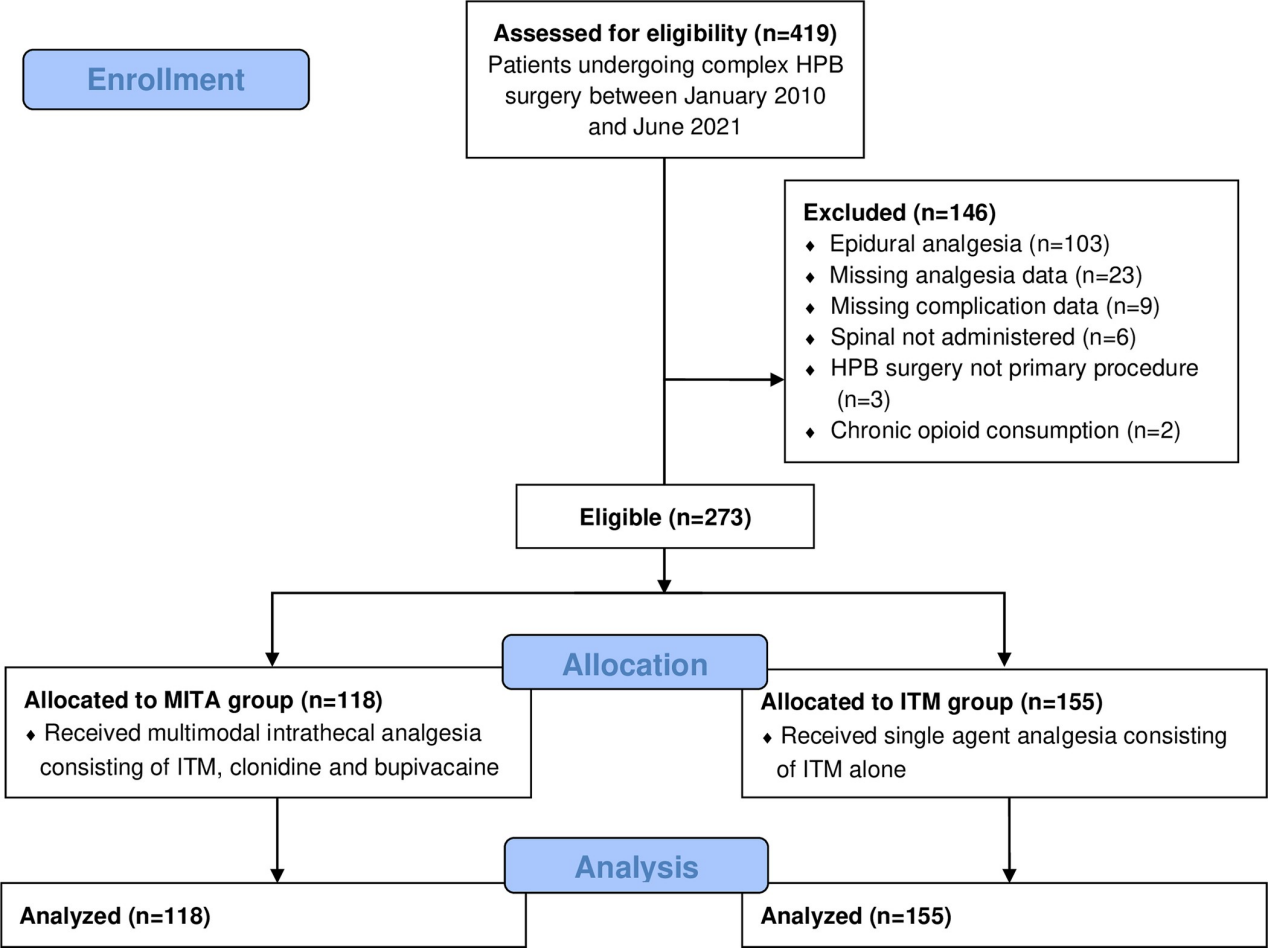
Flow diagram.

During the study period, 419 patients underwent complex HPB surgery, of whom 146 patients were excluded (Fig 1). The number of patients excluded is summarized in a flow diagram (Fig 1). Two hundred and seventy-three patients met the inclusion criteria, of which 118 (43%) received MITA (MITA group) and 155 (57%) received ITM alone (ITM group). 30 (11%) patients underwent surgery at Warringal Private Hospital (Hospital A), 42 (15%) at Knox Private Hospital (Hospital B), and 201 (74%) at Austin Hospital (Hospital C). The baseline patient characteristics and differences in preoperative variables are presented in Table 1.

**Table 1:** Baseline patient characteristics. Data presented as a number (proportion) or median [interquartile range].

Patient Characteristic	MITA group (n = 118)	ITM group (n = 155)	P Value
Age (years)	66.00 [57.00:72.75]	63.00 [54.50:71.00]	0.182
Hospital A Hospital B Hospital C	30 (25.42%) 42 (35.59%) 46 (38.98%)	0 (0.00%) 0 (0.00%) 155 (100.00%)	<0.001
**Operation Group** Liver resection major* Liver resection minor^#^ Pancreatic resection major^^^ Pancreatic resection minor^^^^	47 (39.83%) 20 (16.95%) 30 (25.42%) 21 (17.80%)	39 (25.16%) 80 (51.61%) 36 (23.23%) 0 (0.00%)	<0.001
**Sex** Male Female	57 (48.31%) 61 (51.69%)	96 (61.94%) 59 (38.06%)	0.027
Body mass index (kg/m^2^)	25.31 [22.32:31.08]	26.13 [23.06:29.96]	0.607
**Preoperative Bloods** Hemoglobin (g/L) White cell count (x10^9^/L) Platelets (x10^9^/L) Urea (mmol/L) eGFR (mL/min/1.73 m^2^) Creatinine (μmol/L) Albumin (g/L) Bilirubin (μmol/L) Aspartate transaminase (U/L) Alanine transaminase (U/L) Alkaline phosphatase (U/L) Gamma-glutamyl transferase (U/L) International Normalized Ratio Prothrombin time (sec) Activated partial thromboplastin time (sec)	131.00 [116.75:144.00] 7.50 [5.58:10.13] 234.50 [181.75:319.25] 6.00 [4.90:7.40] 89.00 [70.50:>90.00] 73.00 [61.00:88.00] 38.00 [35.00:41.00] 10.00 [6.00:15.00] 36.50 [24.50:80.50] 36.00 [22.00:77.00] 95.00 [67.00:134.00] 56.00 [36.00:116.25] 1.00 [1.00:1.10] 11.90 [11.00:12.70] 30.00 [27.00:33.75]	141.00 [129.00:149.00] 6.90 [5.70:8.80] 218.00 [160.00:280.00] 5.60 [4.50:7.15] 88.00 [78.25:>90.00] 72.50 [62.25:84.00] 37.00 [32.00:40.00] 13.00 [8.00:38.00] 53.00 [47.00:60.50] 49.00 [38.00:96.00] 145.00 [127.00:202.75] 127.00 [71.00:188.50] 1.10 [1.00:1.10] 31.00 [31.00:31.00] 27.00 [25.00:29.00]	0.001 0.221 0.076 0.072 0.306 0.938 0.177 0.004 0.383 0.058 0.010 0.007 0.007 0.095 <0.001

* Resection of ≥4 segments

^#^ Resection of <4 segments

^^^ Whipple procedure or central or total pancreatectomy

^^^^ Distal pancreatectomy with or without splenectomy, Hospital A = Warringal Private Hospital, Hospital B = Knox Private Hospital, and Hospital C = Austin Hospital.

### Intraoperative data

Detailed intraoperative data, including fluid use, blood loss, and vasoactive medication use, are summarized in Table 2. The median doses of ITM, clonidine, and bupivacaine in the MITA group were 400 μg (300:400), 75 μg (75:100), and 15 mg (15:15), respectively. The median (IQR) dose of ITM in the ITM group was 300 μg (250:400).

**Table 2:** Intraoperative characteristics. Data presented as a number (proportion) or median [interquartile range.

Patient Characteristic	MITA group (n = 118)	ITM group (n = 155)	P Value
Operation time (min)	405.00 [300.00:532.50]	307.00 [226.00:409.25]	<0.001
Intrathecal morphine dose (μg)	400.00 [300.00:400.00]	300.00 [250.00:400.00]	<0.001
Intrathecal clonidine dose (μg)	75 [75:100]	N/A	N/A
Intrathecal bupivacaine dose (mg)	15.00 [15.00:15.00]	N/A	N/A
Estimated blood loss (mL)	300.00 [200.00:400.00]	150.00 [150.00:300.00]	0.003
Urine output (mL)	600.00 [300.00:790.00]	300.00 [300.00:356.25]	<0.001
**Crystalloid solution** No. of patients Amount administered (mL)	118 (100%) 1000.00 [1000.00:1000.00]	155 (100%) 3000.00 [2000.00:3750.00]	>0.999 <0.001
**4% Albumin** No. of patients Amount administered (mL)	53 (44.92%) 500.00 [500.00:1000.00]	49 (31.61%) 500.00 [200.00:500.00]	0.034 <0.001
**20% Albumin** No. of patients receiving Total dose administered (mL)	33 (27.97%) 200.00 [200.00:200.00]	37 (23.87%) 200.00 [200.00:400.00]	0.530 0.928
**Norepinephrine** No. of patients Total dose administered (*u*g)	43 (36.44%) 1080.00 [615.00:2580.00]	4 (2.58%) 160.00 [19.50:345.00]	<0.001 0.005
**Ephedrine** No. of patients Total dose administered (mg)	12 (10.17%) 27.50 [13.75:30.00]	4 (2.58%) 4.50 [3.00:7.50]	0.010 0.014
**Intraoperative IV opioid use (excluding ITM)** No. of patients Intraoperative IV morphine equivalent (mg)	51 (43.22%) 10.00 [10.00: 20.00]	146 (94.19%) 35.00 [25.42:50.00]	<0.001 <0.001

In total, 51 patients (43.2%) in the MITA group received additional intraoperative opioid analgesia compared with 146 patients (94.1%) in the ITM group (P < 0.001). The median intraoperative dose of IV opioid, expressed in IV morphine equivalent was 10 mg (10:20) in the MITA group and 35 mg (25.4:50) in the ITM group (P < 0.001).

### Postoperative oMEDD use and pain scores

**Fig 2:**
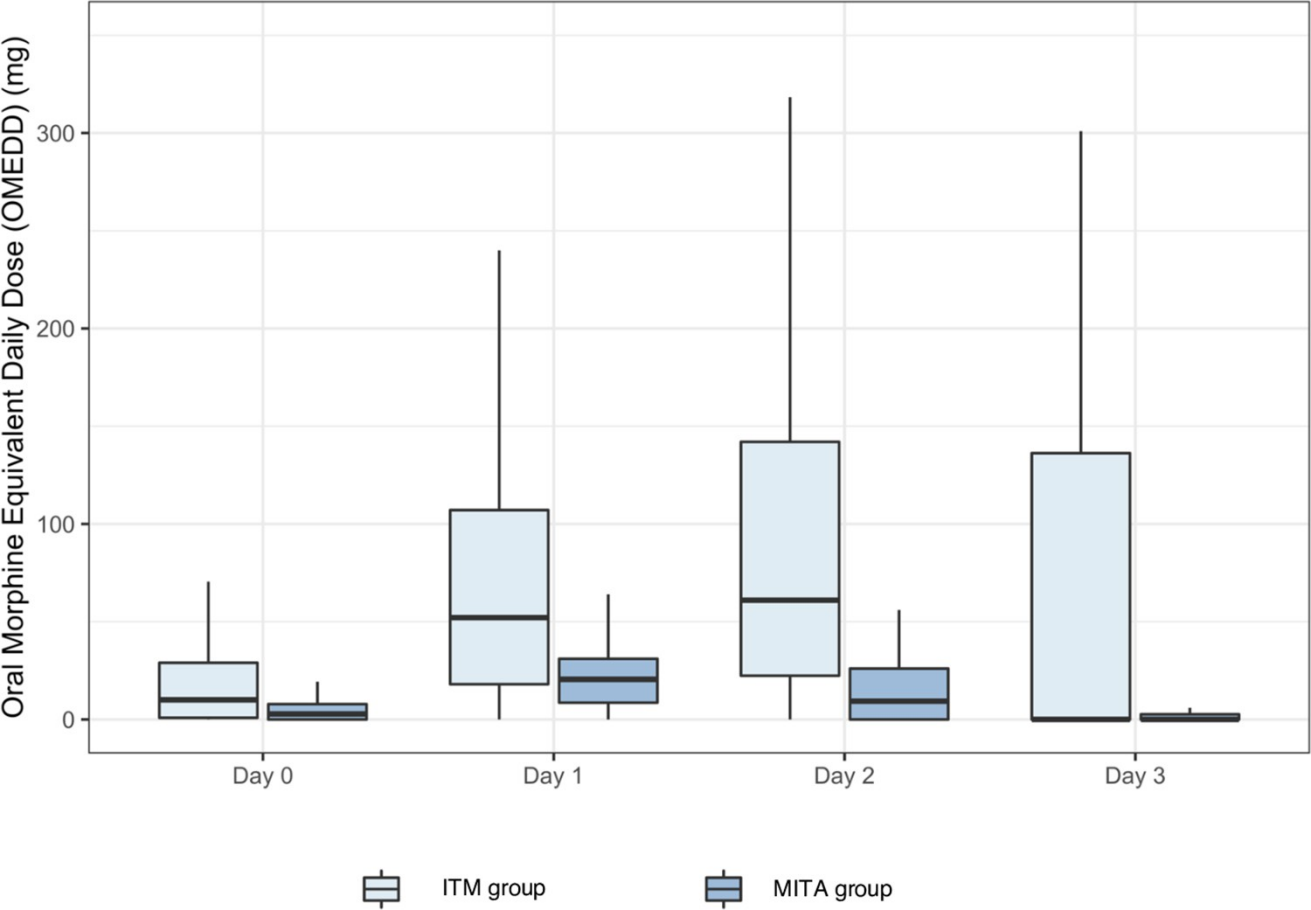
Postoperative oMEDD use.

The median (IQR) cumulative oMEDD use on postoperative day 1 was 20.5 mg (8.6:31.0) in the MITA group compared to 52.1 mg (18.0:107.0) in the ITM group (P < 0.001). This reduction in oMEDD use was observed throughout the entire postoperative period. Postoperative oMEDD use is shown in Fig 2 and Table 3. Patients in the MITA group had lower maximum pain scores on movement and at rest on postoperative days 1–3 (Table 3**)**.

**Table 3:** Postoperative oMEDD (mg) use and maximum pain scores at rest and movement. Data presented as a median [interquartile range].

	Day 0 (day of surgery)	Day 1	Day 2	Day 3
**oMEDD (mg)**				
ITM group	10.00 [0.84:29.00]	52.10 [18.00:107.00]	61.00 [22.30:142.00]	0.00 [0.00:136.00]
MITA group	2.8 [0.00:7.80]	20.50 [8.56: 31.00]	9.33 [0.00:23.00]	0.00 [0.00:2.60]
P Value	<0.001	<0.001	<0.001	<0.001
**Maximum pain scores–on movement**				
ITM group	5.00 [2.00:7.00]	6.00 [4.00:8.00]	6.00 [4.00:8.00]	5.00 [5.00:6.00]
MITA group	3.00 [3.00:3:00]	5.00 [5.00:6.00]	4.00 [4.00:5.00]	4.00 [4.00:4.00]
P Value	0.001	0.002	<0.001	<0.001
**Maximum pain scores–at rest**				
ITM group	3.00 [1.00:5.00]	4.00 [2.00:6.00]	4.00 [2.00:5.00]	3.00 [2.00:4.00]
MITA group	0.00 [0.00:2.75]	3.00 [0.00:5.00]	3.00 [1.00:3.00]	2.00 [1.00:2.00]
P Value	<0.001	0.001	<0.001	<0.001

After adjusting for age, body mass index, hospital allocation, type of surgery, operation length, and intraoperative opioid use, the difference in oMEDD use between the groups was determined at each percentile (Table 4). For the MITA group, on postoperative day 1, patients in the 25^th^ percentile required 14.0 mg less oMEDD (95% CI: -25.9 to -2.2; P = 0.025), patients in the 50^th^ percentile required 27.8 mg less oMEDD (95% CI: -49.7 to -6.0; P = 0.005), and patients in the 75^th^ percentile required 38.7 mg less oMEDD (95% CI: -72.2 to -5.1; P = 0.041).

**Table 4:** Adjusted OMEDD (mg). Data presented as quartile differences (95% confidence interval) for oMEDD between the Multimodal group and the ITM group. Adjusted for patient age, BMI, hospital, surgery type, operation length, and intraoperative opioid use.

	Day 0					
Covariate	25^th^ Percentile	P-value	50^th^ Percentile	P-value	75^th^ Percentile	P-value
**MITA group** **ITM group**	-1.670 [-4.353:1.013] (Reference)	0.234	-4.919 [-10.663:0.824] (Reference)	0.068	-11.086 [-20.602:-1.571] (Reference)	0.014
**Age (years)**	-0.118 [-0.230:-0.006]	0.051	-0.359 [-0.529:-0.189]	<0.001	-0.340 [-0.537:-0.142]	0.001
**BMI (kg/m** ^**2**^ **)**	0.039 [-0.107:0.186]	0.606	-0.060 [-0.361:0.240]	0.682	-0.193 [-0.671:0.285]	0.408
**Hospital A** **Hospital B** **Hospital C**	(Reference) 1.361 [-1.590:4.311] -0.401 [-3.120:2.318]	0.359 0.778	(Reference) 1.807 [-3.850:7.464] -1.568 [-7.010:3.874]	0.524 0.546	(Reference) -0.701 [-8.416:7.015] -2.287 [-10.428:5.854]	0.860 0.490
**Major liver resection** **Minor liver resection** **Major pancreatic resection** **Minor pancreatic resection**	(Reference) 0.773 [-1.584:3.129] 1.813 [-0.602:4.227] 1.008 [-1.557:3.573]	0.480 0.137 0.416	(Reference) 4.715 [-1.992:11.422] 3.565 [-1.553:8.684] 3.804 [-1.591:9.198]	0.192 0.229 0.218	(Reference) 5.398 [-4.119:14.915] 1.423 [-7.260:10.106] -0.239 [-7.436:6.959]	0.228 0.706 0.946
**Operation length (min)**	-0.001 [-0.008:0.006]	0.807	-0.005 [-0.018:0.009]	0.502	-0.008 [-0.028:0.012]	0.431
**Intraoperative IV morphine equivalent**	-0.009 [-0.075:0.058]	0.784	0.104 [-0.069:0.278]	0.250	0.164 [-0.043:0.372]	0.147
	**Day 1 (primary outcome)**					
	**25% Percentile**	**P-value**	**50% Percentile**	**P-value**	**75% Percentile**	**P-value**
**MITA group** **ITM group**	-14.041[-25.853:-2.229] (Reference)	0.025	-27.846 [-49.668:-6.025] (Reference)	0.005	-38.674 [-72.230:-5.119] (Reference)	0.041
**Age (years)**	-0.558 [-1.000:-0.116]	0.007	-0.732 [-1.445:-0.020]	0.040	-1.029 [-1.950:-0.108]	0.042
**BMI (kg/m** ^**2**^ **)**	-0.127 [-0.699:0.445]	0.730	-0.755 [-1.844:0.334]	0.183	-0.610 [-1.983:0.763]	0.412
**Hospital A** **Hospital B** **Hospital C**	(Reference) 14.214 [-2.549:30.977] -3.731 [-18.424:10.961]	0.084 0.578	(Reference) 6.078 [-12.611:24.768] -9.182 [-24.334:5.970]	0.493 0.279	(Reference) 6.086 [-17.722:29.895] -9.156 [-30.202:11.891]	0.576 0.351
**Major liver resection** **Minor liver resection** **Major pancreatic resection** **Minor pancreatic resection**	(Reference) 1.160 [-12.125:14.445] 7.327 [-6.075:20.729] 3.416 [-7.452:14.283]	0.859 0.264 0.549	(Reference) 0.001 [-19.962:19.965] 6.251 [-8.161:20.663] -0.308[-14.563:13.946]	>0.999 0.403 0.968	(Reference) 12.009 [-21.543:45.561] -2.289 [-27.381:22.803] 1.659 [-22.325:19.007]	0.492 0.846 0.875
**Operation length (min)**	-0.015 [-0.045:0.016]	0.291	-0.025 [-0.061:0.012]	0.141	-0.024 [-0.079:0.030]	0.340
**Intraoperative IV morphine equivalent**	0.024 [-0.309:0.356]	0.878	0.346 [-0.310:1.002]	0.224	0.987 [-0.062:2.035]	0.095
	**Day 2**					
	**25% Percentile**	**P-value**	**50% Percentile**	**P-value**	**75% Percentile**	**P-value**
**MITA group** **ITM group**	-29.058 [-46.780:-11.335] (Reference)	0.001	-53.184 [-74.857:-31.511] (Reference)	<0.001	-60.894 [-103.635:-18.152] (Reference)	0.010
**Age (years)**	-0.148 [-0.591:0.295]	0.480	-0.721 [-1.714:0.273]	0.121	-2.008 [-3.394:-0.622]	0.008
**BMI (kg/m** ^**2**^ **)**	0.090 [-0.542:0.722]	0.784	-0.555 [-1.580:0.470]	0.356	-0.958 [-3.061:0.1.146]	0.392
**Hospital A** **Hospital B** **Hospital C**	(Reference) 1.616 [-10.856:14.088] -1.672 [-9.234:5.890]	0.808 0.641	(Reference) 4.608 [-14.690:23.905] -0.358 [-15.268:14.552]	0.617 0.960	(Reference) 1.226 [-28.779:31.232] -13.434 [-45.787:18.919]	0.938 0.382
**Major liver resection** **Minor liver resection** **Major pancreatic resection** **Minor pancreatic resection**	(Reference) 3.236 [-9.121:15.592] -0.200 [-13.574:13.174] 2.437 [-6.296:11.169]	0.536 0.976 0.530	(Reference) 11.227 [-17.616:40.069] 2.654 [-16.732:22.039] 7.226 [-8.127:22.579]	0.422 0.783 0.387	(Reference) 45.702 [-5.939:97.342] -7.150 [-33.119:18.818] 9.569 [-19.815:38.953]	0.083 0.548 0.552
**Operation length (min)**	0.010 [-0.018:0.038]	0.467	0.008 [-0.039:0.055]	0.727	-0.007 [-0.074:0.060]	0.843
**Intraoperative IV morphine equivalent**	-0.132 [-0.449:0.184]	0.467	-0.005 [-0.737:0.727]	0.990	1.181 [-0.175:2.537]	0.129

### Opioid-related adverse events and complications

The incidence of postoperative respiratory depression requiring naloxone was low (<1.5%) and similar between the groups. Hypotension requiring vasopressor support occurred more frequently in the MITA group on postoperative day 0 and persisted through to postoperative day 3 **(**Table 5**).** There was a higher incidence of minor complications in the MITA group, but there were no significant differences in the development of major complications between the groups **(**Table 6**).** The median (IQR) length of hospital stay in the MITA group was 9 days (6.0:15.0), compared to 8 days (6.0:11.5) in the ITM group (P = 0.203). The length of hospital stays across the different types of surgery are summarized in Table 6.

**Table 5:** Opioid-related adverse events. Data presented as number of patients (proportion) or median [interquartile range].

Patient characteristic	MITA group (n = 118)	ITM group (n = 155)	P Value
**Respiratory depression requiring naloxone** Day 0 Day 1	1 (0.85%) 0 (0.00%)	2 (1.29%) 1 (0.65%)	0.412 0.252
**Hypotension requiring vasopressor support** Day 0 Day 1 Day 2 Day 3	50 (42.37%) 50 (42.37%) 29 (24.58%) 12 (10.17%)	29 (18.71%) 8 (5.16%) 3 (1.94%) 4 (2.58%)	<0.001 <0.001 <0.001 0.010
**Pruritis (any postoperative day)**	12 (10.17%)	17 (10.97%)	>0.999
**Nausea or vomiting (any postoperative day)**	14 (11.86%)	23 (14.84%)	0.466
**Highest sedation score**^**#**^ **(day 0)**	1.00 [0.00:1.00]	1.00 [1.00:2.00]	<0.001

^#^ Sedation score:0 = awake and alert, 1 = occasionally drowsy, easily roused, 2 = often drowsy, easily roused with touch, 3 = somnolent, difficult to rouse.

**Table 6:** Major adverse events. Data presented as number of patients (proportion) or median [interquartile range].

	MITA group (n = 118)	ITM group (n = 155)	P Value
**Any postoperative complication**	88 (74.58%)	113 (72.90%)	0.757
**Number of complications per patient**	1.00 [0.25:2.00]	2.00 [0.00:4.00]	0.071
**Number of complications** 0 complication 1 complication 2 complications 3 complications 4+ complications	30 (25.42%) 36 (30.51%) 24 (20.34%) 15 (12.71%) 13 (11.02%)	42 (27.10%) 30 (19.35%) 25 (16.13%) 17 (10.97%) 41 (26.45%)	0.757 0.033 0.371 0.659 0.002
**Worst complication (Clavien-Dindo classification)** No complication Grade I Grade II Grade III Grade IV Grade V	30 (25.42%) 46 (38.98%) 36 (30.51%) 5 (4.24%) 1 (0.85%) 0 (0.00%)	42 (27.10%) 30 (19.35%) 67 (43.23%) 8 (5.16%) 7 (4.52%) 1 (0.65%)	0.046 0.757 <0.001 0.032 0.725 0.076 0.387
**Length of hospital stay (days)** All patients Liver resection major* Liver resection minor^#^ Pancreatic resection major^^^ Pancreatic resection minor^^^^	9.00 [6.00:15.00] 7.00 [6.00:10.50] 6.00 [4.50:8.50] 13.50 [9.25:18.50] 9.00 [8.00:17.00]	8.00 [6.00:11.50] 8.00 [6.00:13.50] 6.00 [5.00:8.00] 11.00 [9.00:17.25] N/A	0.203 0.172 0.238 0.506 N/A
**Intensive care unit admission**	118 (100.00%)	142 (91.61%)	<0.001
**Intensive care unit stay (days)**	2.00 [1.00:3.00]	1.00 [1.00:1.00]	<0.001
**Type of complication**			
**Cardiovascular** Arrhythmia Congestive cardiac failure Myocardial infarction Other cardiovascular	10 (8.37%) 3 (2.54%) 1 (0.85%) 1 (0.85%)	20 (12.90%) 21 (13.54%) 0 (0.00%) 2 (1.29%)	0.248 0.002 0.255 0.732
**Respiratory** Atelectasis Pneumonia Pleural effusion Pulmonary embolus Respiratory failure Other respiratory	26 (22.03%) 5 (4.24%) 6 (5.08%) 2 (1.69%) 3 (2.54%) 1 (0.85%)	23 (14.84%) 16 (10.32%) 10 (6.45%) 4 (2.58%) 10 (6.45%) 5 (3.23%)	0.126 0.062 0.636 0.624 0.134 0.186
**Gastrointestinal** Postoperative pancreatic fistula Nausea and vomiting Constipation Diarrhea Ileus Surgical site infection Gastrointestinal bleed Liver failure Other gastrointestinal	1 (0.85%) 20 (16.95%) 4 (3.39%) 3 2.54%) 7 (5.93%) 6 (5.08%) 0 (0.00%) 1 (0.85%) 2 (1.69%)	6 (3.87%) 17 (10.97%) 3 (1.93%) 4 (2.58%) 22 (14.19%) 21 (13.55%) 4 (2.58%) 4 (2.58%) 13 (8.39%)	0.119 0.154 0.454 0.986 0.029 0.021 0.080 0.292 0.017
**Hematological** Anemia requiring treatment Thrombosis Hemorrhage Other hematological	8 (6.78%) 3 (2.54%) 1 (0.85%) 1 (0.85%)	23 (14.84%) 2 (1.29%) 4 (2.58%) 4 (2.58%)	0.038 0.448 0.292 0.292
**Genitourinary** Acute kidney injury Urinary tract infection Other genitourinary	13 (11.02%) 0 (0.00%) 2 (1.69%)	12 (7.74%) 1 (0.65%) 5 (3.23%)	0.354 0.387 0.430
**Endocrine** Dysglycemia Any electrolyte derangement Hypokalemia Hyponatremia Hypomagnesemia Hypophosphatemia Hypocalcemia Hyperkalemia Hypernatremia Other endocrine	8 (6.78%) 19 (16.10%) 12 (10.17%) 1 (0.85%) 0 (0.00%) 4 (4.49%) 1 (0.85%) 1 (0.85%) 0 (0.00%) 0 (0.00%)	6 (3.87%) 27 (17.42%) 15 (9.68%) 1 (0.65%) 1 (0.65% 2 (1.29%) 0 (0.00%) 4 (2.58%) 2 (1.29%) 2 (1.29%)	0.282 0.775 0.894 0.850 0.387 0.243 0.255 0.292 0.218 0.218
**Other** Delirium Postoperative stroke/transient ischemic attack Postoperative systemic inflammatory response syndrome Dermatological Death Other	2 (1.69%) 1 (0.85%) 8 (6.78%) 1 (0.85%) 0 (0.00%) 5 (4.24%)	10 (6.45%) 1 (0.65%) 10 (6.45%) 3 (1.94%) 1 (0.65%) 27 (17.42%)	0.058 0.850 0.915 0.462 0.387 <0.001

* Resection of ≥4 segments

^#^ Resection of <4 segments

^^^ Whipple procedure or central or total pancreatectomy

^^^^ Distal pancreatectomy with or without splenectomy; ^#^ Sedation score: 0 = awake and alert, 1 = occasionally drowsy, easily roused, 2 = often drowsy, easily roused with touch, 3 = somnolent, difficult to rouse.

## Discussion

### Key findings

We conducted a multicenter retrospective observational study in patients undergoing complex abdominal surgery comparing the analgesic effects of a multimodal combination of ITM, clonidine, and bupivacaine against ITM alone. As hypothesized, multimodal intrathecal analgesia (MITA) significantly reduced postoperative opioid use and resulted in superior postoperative analgesia without increased risks of respiratory depression. The improved postoperative analgesia and reduced oMEDD use extending into the postoperative period may be explained by the higher dose of ITM administered to the MITA group in addition to the analgesic effects of intrathecal clonidine and bupivacaine. However, these analgesic benefits extended to patient sub-cohorts at the 25^th^ and 75^th^ quartiles. These findings are consistent with other studies that support the use of multiple-agent intrathecal analgesia techniques using ITM [12,14].

### Postulated mechanisms

We acknowledge that the improved postoperative analgesia and reduced oMEDD use extending into the postoperative period may be explained by the higher dose of ITM administered to the MITA group. However, these findings may also be attributable to the synergistic pharmacokinetic and pharmacodynamic effects of the distinctive mechanisms of action of the individual constituents present in MITA. The concurrent mechanism of actions of ITM, bupivacaine and clonidine are complementary, allowing for enhanced analgesia while minimizing adverse effects.

Intrathecal morphine binds to opioid receptors that are coupled to G-proteins in the dorsal horn neurons, which in turn mediate the activation of potassium channels and the inhibition of calcium channels [24,25]. Morphine’s hydrophilic nature and lipid solubility confines its distribution primarily within the cerebrospinal fluid compartment around the spinal cord, thus leading to localized and prolonged effects. Further, the activation of mu-opioid receptors decreases intracellular calcium levels leading to a reduction in the excitation of presynaptic C fibers and modulating descending inhibitory pathways, culminating in diminished nociception.

Conversely, the synergistic effects of intrathecal bupivacaine arise from its rapid diffusion to the nerve roots and spinal cord tissues, blocking voltage-gated channels, inhibiting nerve conduction, and leading to rapid and reliable onset of anesthesia and analgesia. Due to its limited systemic distribution, bupivacaine also remains localized to the spinal area, where it exerts analgesic effects by blocking voltage-gated sodium channels on neuronal cell membranes [26,27]. This blockade reduces the influx of sodium into the cells, raising the threshold for electrical excitation and diminishing the generation of action potentials and conduction of nerve impulses [26–28]. Bupivacaine’s sensory blockade reduces afferent nociceptive input, thereby amplifying morphine impact on modulating central pain pathways. Accordingly, the combination of bupivacaine with morphine enhances analgesia and exploits their divergent mechanisms of action through the creation of a synergistic effect. The net effect of their co-administration is an additive postoperative analgesic benefit, achieved at lower doses of each drug.

The concomitant administration of intrathecal clonidine further enhances the effects of ITM and intrathecal bupivacaine, as clonidine not only prolongs analgesia but mitigates the potential for rebound hyperalgesia. These effects are thought to be mediated through the modulation of pathways in the dorsal horn, resulting in a reduction in sympathetic outflow [17,29]. The effectiveness of intrathecal bupivacaine is influenced by its dose-response relationship and several investigations have reported a duration of action lasting from 1.5 to 3 hours [30,31]. However, the addition of intrathecal clonidine increases bupivacaine’s duration of action by 116% [31]. Similar to our findings, others have reported that patients who were administered both these agents experienced significantly reduced pain scores and longer analgesia within a 24-hour period following orthopedic surgery [32]. In addition, intrathecal clonidine prolonged the analgesic effects of intrathecal bupivacaine in gynecological surgeries [33,34]. Intrathecal clonidine therefore enhances the efficacy and duration of analgesia when combined with local anesthetic or opioid solutions [29,35].

The inherent characteristics of morphine combined with the pharmacodynamic interactions of bupivacaine and clonidine, collectively contribute to prolonged postoperative analgesia following intrathecal administration. Therefore, our findings suggest that the utilization of MITA for patients undergoing major abdominal surgery presents several advantages compared to ITM alone. These include heightened and extended analgesia from the combined and synergistic mechanisms of action, diminished occurrence of systemic adverse effects due to each medication’s primary impact within the spinal cord, and enhanced postoperative analgesia.

### Clinical implications

The optimal dose of ITM for postoperative analgesia is unclear, and dose-response curves in the setting of complex abdominal surgery are not available in the literature. In our study, the doses of morphine in the MITA and ITM groups were 400 μg and 300 μg, respectively. Our findings suggest that an ITM dose of 400 μg (combined with clonidine and bupivacaine), when compared to ITM monotherapy at a dose of 300 μg, results in superior postoperative analgesia over a longer postoperative duration without any adverse effects on respiratory depression or major complications. Our findings reinforce the respiratory depression safety profile of this specific dosing strategy. Our findings further imply that the effects of intrathecal morphine are primarily localized to the spinal cord and do not significantly affect supraspinal opioid receptors, minimizing systemic side effects commonly associated with opioids. By directly targeting the spinal cord opioid receptors, intrathecal morphine may provide superior analgesia compared to systemic opioids alone for patients undergoing major HPB surgery. However, our findings also imply that a dose of 400 μg of ITM in combination with intrathecal clonidine and bupivacaine is associated with a greater incidence of hypotension requiring vasopressor support on postoperative days 0 to 3.

### Relationship to the literature

Intrathecal morphine has been recommended as the gold standard in intraoperative analgesia by the ERAS society [9]. However, the peak analgesic effect of ITM takes approximately 6 hours from the time of administration [13]. We predicted that patients who received intrathecal bupivacaine would receive less supplemental opioid analgesia by acting as a bridging agent until ITM reached optimal effectiveness. As recommended in the literature, multimodal analgesia is the mainstay in open HPB surgery [36].

Nguyen et al. reported that the addition of bupivacaine resulted in lower supplemental opioid use in patients undergoing liver resection surgery [12], findings that were also consistent with studies evaluating analgesia outcomes in patients undergoing biliary, colorectal, and urological surgeries [37–39] Intraoperative MITA has been shown to provide pain relief in the early postoperative period [12]. Some studies have reported that the use of ITM results in improvements in pain scores only in the first 24 hours post-surgery [40–42]. However, our findings demonstrate that the duration of the ITM effect, when combined with bupivacaine and clonidine, extends through to at least the second and third postoperative days. In the present study, although MITA was not associated with postoperative respiratory depression or excess sedation compared to ITM alone, we observed a significantly higher incidence of postoperative hypotension, most likely attributed by the sympathetic blockade and additional vasodilating effects of clonidine and bupivacaine. Such findings are also reported in other studies [12,43,44].

Our study expands upon the existing literature and provides consideration as to the magnitude and efficacy of using MITA by considering sub-cohorts at different quartiles. Most studies in the literature that investigate analgesic interventions only compare the ‘average’ response between cohorts, usually at the 50^th^ percentile (median) or mean. However, there are numerous factors that contribute to variations in opioid use in the postoperative period. Patients who generally require high doses of opioids (i.e., are in the higher quartile of the population) to achieve satisfactory analgesic outcomes may not see the same magnitude of impact as those who may require far lower doses of opioids. Therefore, it follows that alternative analgesic interventions may have varied results for patients depending on their ‘position’ within a cohort. Our study provides consideration around this and allows further studies to also consider this when evaluating potential interventions.

Kong et al. administered 200 μg of ITM in patients undergoing colorectal surgery without any significant adverse effects, and doses as high as 500 μg have been used safely in open liver resection surgery [37,45]. A dose reduction in ITM may be warranted in patients at higher risk of respiratory depression, such as those with severe chronic pulmonary disease or severe obstructive sleep apnea [45].

A consensus regarding the ideal intrathecal bupivacaine dosage is also not available. It has been shown that an intrathecal dose of 7.5mg can provide optimal analgesia and allow for day procedures aiming at same-day discharge [46]. However, larger doses required for more invasive and complex surgical procedures require doses upward of 15mg, as used in the MITA group. Increasing doses are associated with higher incidences of adverse events and hemodynamic disturbances, in particular hypotension [47]. Larger doses of bupivacaine could also influence postoperative blood pressures, resulting in hypotensive events requiring intervention. This impact was observed in our cohort, as the rate of hypotensive events requiring vasopressor support was significantly higher in the MITA group compared to the ITM group. Injection of all three agents from the MITA regimen into the intrathecal space ensures efficiency and convenience. However, it has been shown that introducing bupivacaine into the epidural space rather than the spinal cavity could avoid deep sympathetic block and promote more hemodynamic stability [48].

In our study, we used hyperbaric bupivacaine on all patients in the MITA group. Immediately after its administration, we deliberatively positioned all patients in a steep Trendelenburg position with flexion of the hips to increase the cephalad spread to affect the thoracic spinal nerves, while increasing preload to minimize the hypotensive effects of these medications. Nevertheless, the addition of clonidine and bupivacaine contributed to the higher incidence of intra- and postoperative hypotension. Therefore, due to potential hemodynamic instability, the use of spinal analgesia in the setting of general anesthesia should be done so with caution [49]. This finding reinforces the need for vigilant perioperative blood pressure monitoring and intensive care support to facilitate the delivery of vasoactive medications to treat hypotension if required.

The literature suggests that the use of ITM is associated with higher rates of respiratory depression [50]. However, in our study, the addition of bupivacaine and clonidine to ITM did not significantly affect the rate of respiratory depression requiring naloxone. It has been reported in the literature that respiratory depression is more common when ITM is combined with spinal anesthesia if the dose of ITM is more than 500 μg. These findings were not observed in the present study [51]. Other opioid-related complications were also stable between groups, including pruritis as well as nausea and vomiting. In addition to reducing the doses of ITM and intrathecal bupivacaine required, the use of intrathecal clonidine could potentially have had a protective effect on the side effect profile in the MITA group. In the setting of neuraxial anesthesia, intrathecal clonidine has been reported to be safe in enhancing the analgesic effect through sensory and motor blocks, while also restricting unwanted side effects including pruritis, hypotension, and nausea and vomiting in the postoperative period [52].

### Study strengths and limitations

This study has several strengths. Our study adds to a growing body of literature reporting on the safety of both ITM alone and ITM in combination with other intrathecal analgesics. Even after adjustments for age, body mass index, hospital allocation, operation type, duration of surgery, and intraoperative IV morphine use, our findings show that MITA is associated with less oMEDD use and improved postoperative analgesia. Further, this study expanded the consideration of the ‘average’ magnitude of effect of this intervention and considered the magnitude of effect for other quartiles of the patient population. We demonstrated the efficacy of MITA for those sub-cohorts of patients who have higher or lower opioid requirements in the postoperative period as compared to those considered ‘average’. The relatively large sample size of almost 300 patients further supports our systematic evaluation of clinically meaningful outcomes, such as complications and length of hospital stay.

This study has several limitations that are intrinsic to its retrospective observational study design. Although this study was undertaken in a well-resourced healthcare system in Australia, its external validity is limited to other regions. Our findings are not generalizable to laparoscopic procedures or non-abdominal procedures such as cardiothoracic and orthopedic surgery, nor are they generalizable to pediatric patients. Our study relied on existing data collected, which may have led to potential biases and missing information. We have not been adequately able to control for confounding variables, and there is a risk of unmeasured confounders influencing the observed outcomes. We acknowledge that there were imbalances between the groups. For this reason, as outlined in our statistical analysis, our primary outcomes were adjusted for age, body mass index, hospital allocation, operation type, operation length, and intraoperative IV morphine equivalent. Finally, we acknowledge that we cannot establish a cause-and-effect relationship and are unable to draw definitive conclusions about the effectiveness or safety of MITA. Despite these limitations, our study provides valuable insights and generates hypotheses for further investigation. Our findings should be validated through prospective randomized trials to establish more robust evidence.

## Conclusion

In patients undergoing complex HPB surgery, the use of ITM in combination with intrathecal clonidine and bupivacaine reduced postoperative opioid use and resulted in superior postoperative analgesia without the risk of respiratory depression when compared to patients who received ITM alone. This also applied to patient sub-cohorts at the 25^th^ and 75^th^ quartiles. A randomized prospective clinical trial investigating these two intrathecal analgesic techniques is justified.

## Supporting information

S1 Checklist TREND statement checklist. (PDF)

S2 Checklist STROBE statement—checklist of items that should be included in reports of observational studies. (DOC)

S1 File De-identified database. (XLSX)
